# Isolated Native Tricuspid Valve Endocarditis in an HIV Patient due to Streptococcus Pneumoniae: A Rare Clinical Phenomenon

**DOI:** 10.7759/cureus.10780

**Published:** 2020-10-03

**Authors:** Nouraldeen Manasrah, Roshini Moses, Ali F Al Sbihi, Wasif Hafeez, Arif Hakim

**Affiliations:** 1 Internal Medicine, Detroit Medical Center Sinai-Grace Hospital, Detroit, USA; 2 Cardiology, Beaumont Hospital, Dearborn, USA

**Keywords:** tricuspid valve endocarditis, pneumococcal endocarditis, transesophageal echocardiogram, pneumococcal bacteremia

## Abstract

Streptococcus pneumonia is an important cause of septicemia. Other sites of infection include meningitis, septic arthritis, and endocarditis. Pneumococcal endocarditis is rare and has a poor prognosis. We report a case of a 47-year-old female patient with HIV who developed isolated native tricuspid valve endocarditis secondary to streptococcus pneumonia, which is considered to be a very rare presentation in our patient due to the absence of common risk factors such as intravenous drug use, heart disease, or right heart catheterization.

## Introduction

Pneumococcal bacteremia can occur in patients with or without pneumococcal pneumonia. This bacterium is known to infect both immunocompetent and immunosuppressed individuals. When bacteremia is present, secondary complications, such as arthritis, meningitis, and endocarditis, may occur. Pneumococcal infective endocarditis (IE) is responsible for <1% of IE [1]. Isolated native tricuspid valve endocarditis (TVE) accounts for only 5% to 10% of all cases of IE [2] and is rarely seen in the absence of common risk factors like intravenous drug use (IDU) or congenital heart disease.

## Case presentation

**Figure 1:**
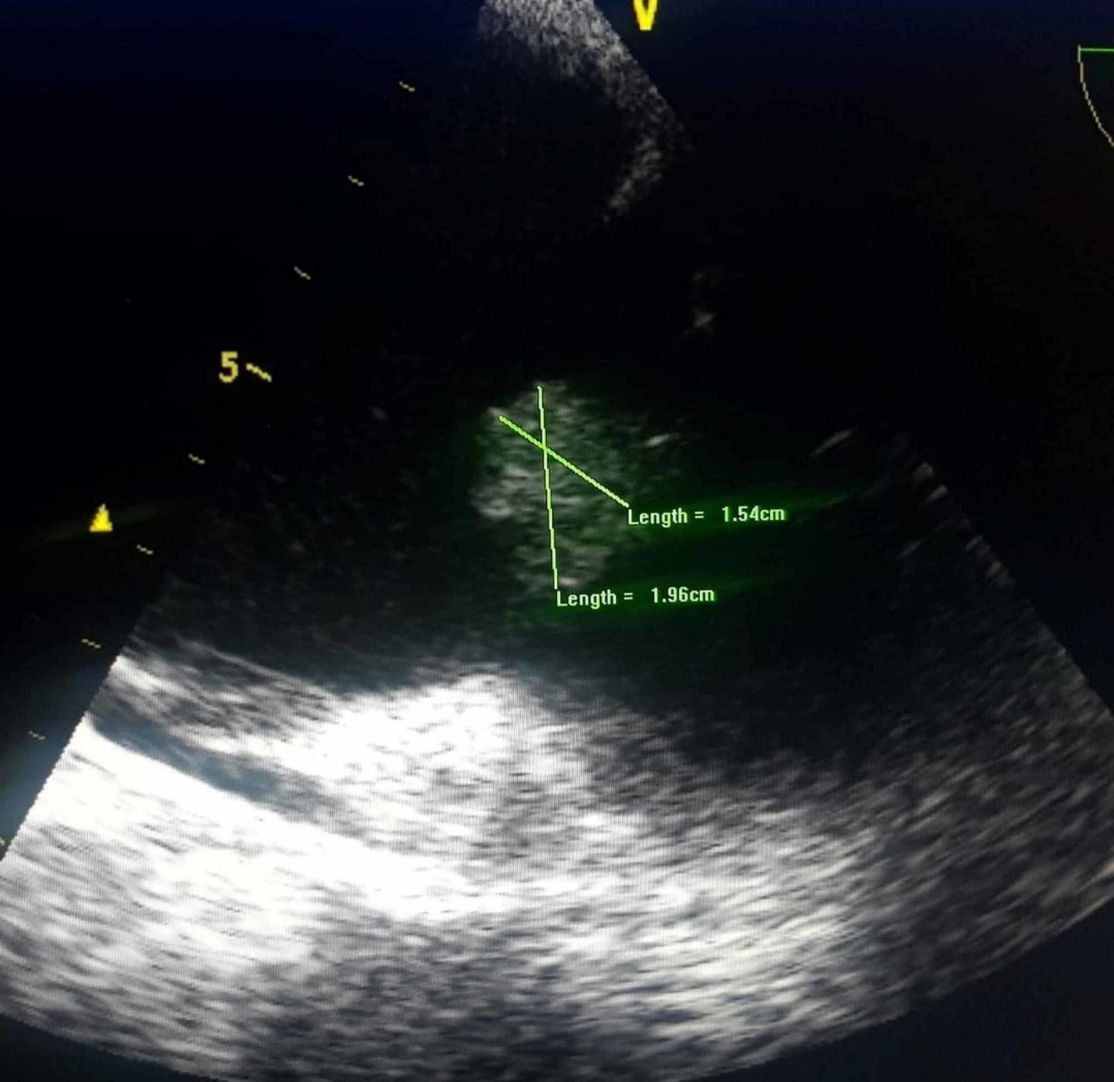
TTE showing 1.9 x 1.5 cm echo density attached to the tricuspid valve leaflet. TTE, transthoracic echocardiography

A 47-year-old African American female presented with complaints of fever, chills, left-sided pleuritic chest pain, and shortness of breath for a month. She denied drug use, extremity swelling, recent travel, or sick contacts. She had been relatively immobile, spending long hours in bed or a chair over the last month. Her past medical history revealed hypertension treated with nifedipine, CKD stage 4, and HIV infection for which she was to be on a combination pill of darunavir/cobicistat/emtricitabine/tenofovir alafenamide (SYMTUZA®), but she had not been compliant lately. She smoked three to four cigarettes per day for the past two years, occasionally used marijuana, and had no history of alcohol or IDU. Her pertinent vital signs on admission were an axillary temperature of 39 degrees Celsius and a heart rate of 110 beats per minute. Cardiac examination showed regular rhythm with normal S1 and S2, and II/VI systolic murmur in the left lower sternal border, which was new compared to the previous admission examination. The respiratory system examination illustrated crackles mainly on the left lower side, and the rest of the examination findings were unremarkable. Laboratory evaluation revealed mild leukocytosis (12.8 k/cumm), thrombocytopenia (70 k/cumm), CD4 counts of 499 cells/mm^3^, HIV viral load of 2,730 copies/mL, and negative urine drug screen. A chest X-ray showed developing left lower lobe pneumonia. She was empirically started on ceftriaxone and azithromycin for community-acquired pneumonia. Two separate sets of blood cultures were positive for *Streptococcus pneumonia* that was pan-sensitive. Lower extremity venous duplex and ventilation-perfusion (V/Q) scan were negative for deep venous thrombus and a low probability of PE, respectively. She did develop consumptive coagulopathy due to sepsis, which improved over her hospital course. On day 6, transthoracic echocardiography (TTE) showed 1.9 x 1.5 cm echo density seemingly attached to the tricuspid valve leaflet, probably posterior leaflet (Figure 1).

**Figure 2:**
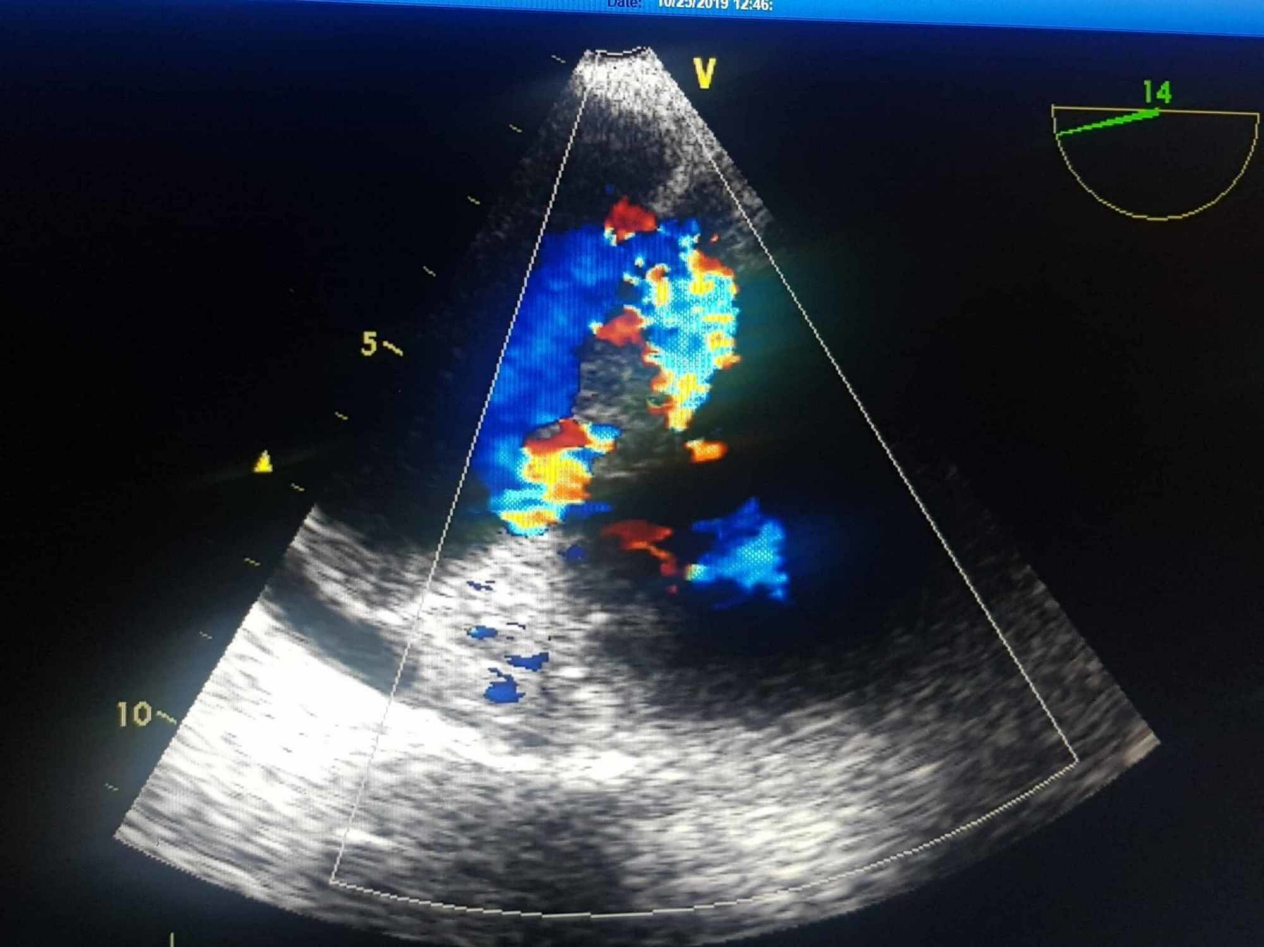
TEE revealing a large tricuspid valve vegetation and a perforated posterior tricuspid leaflet with moderate-to-severe tricuspid valve regurgitation. TEE, transesophageal echocardiography

**Figure 3:**
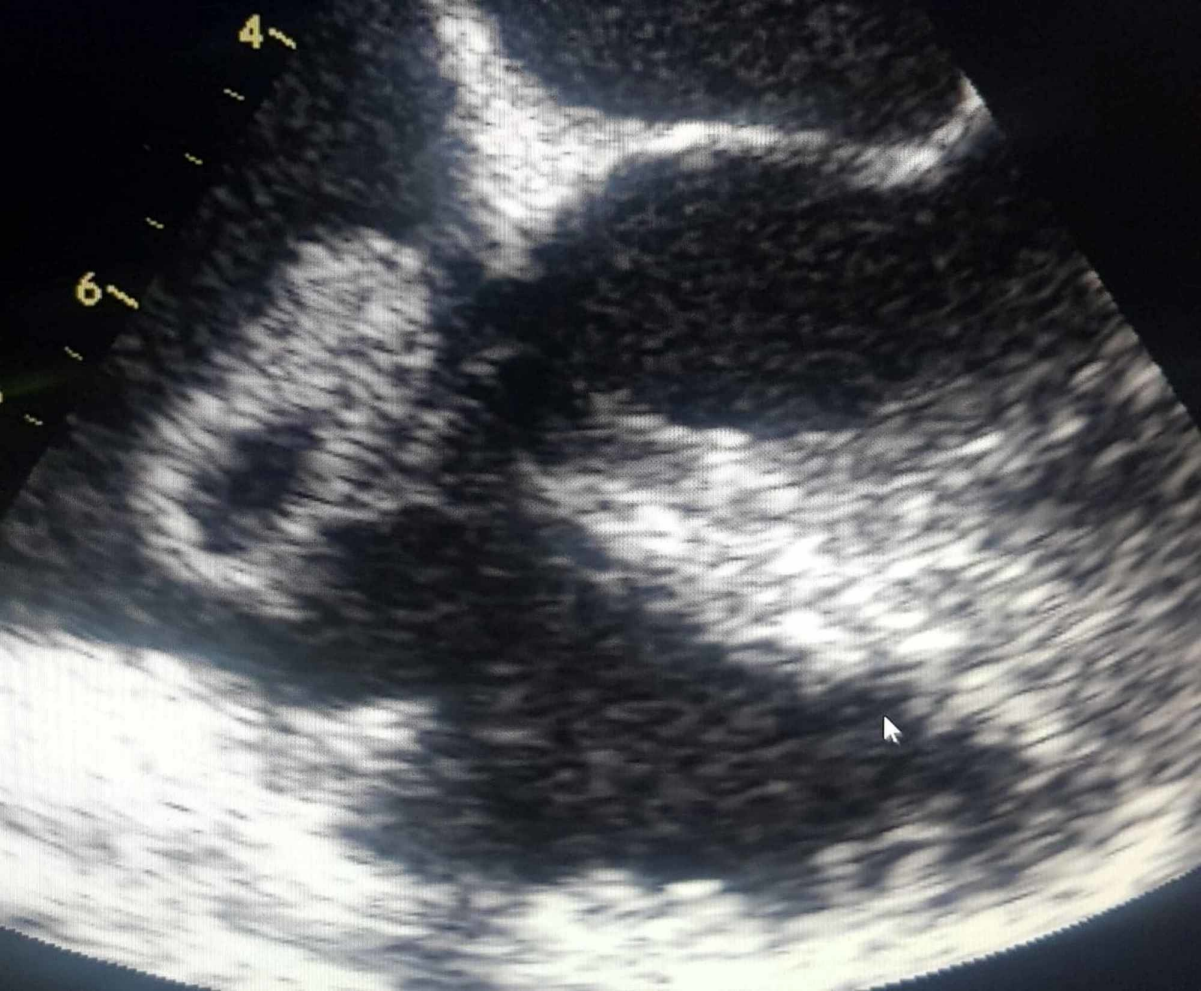
TEE showing large vegetation of tricuspid valve with central liquefaction. TEE, transesophageal echocardiography

The ejection fraction was 55-60%. Transesophageal echocardiography (TEE) on day 8 revealed large tricuspid valve vegetation and a perforated posterior tricuspid leaflet with moderate-to-severe tricuspid valve regurgitation ( Figure 2), with central liquefaction (Figure 3).

The patient refused any surgical intervention for the vegetation and valve repair/replacement. The patient's clinical conditions improved and she was afebrile for 72 hours. She was discharged on ceftriaxone 2 gm to complete a four-week course.

## Discussion

Pneumococcal IE remains rare but carries a poor prognosis. It is responsible for <1% of IE [1]. It has been associated with rapidly progressive IE, extensive valvular destruction, heart failure, and high lethality [3]. Systematic reviews of the literature since 2000 identified 111 cases of pneumococcal IE, which were predominantly left-sided (aortic valve 53.2% and mitral valve 40.5%) [4]. Isolated native TVE represents only 5% to 10% of all cases of IE [2]. The most common predisposing factor for right-sided endocarditis is IDU [5]. Other predisposing factors include intracardiac catheterization, cardiac anomalies, immunodeficiency, and indwelling central venous lines.

What made our case interesting is that our patient had right-sided endocarditis, which is much less frequent than left side, and the fact that she denied IDU. She had no cardiac anomalies as well. Also, Staphylococcus infections are usually the cause of tricuspid valve lesions, with pneumococcus being an uncommon causative organism.

Complications of right-sided endocarditis include pulmonary infarction, pulmonary abscess, progressive right-sided heart failure, and renal abnormalities [6].

Echocardiography is the cornerstone of IE diagnosis. TTE has limitations for the early diagnosis of IE. TEE plays a key diagnostic role, given its greater sensitivity and specificity for detecting valvular vegetations and other possible complications.

A prolonged course of intravenous antibiotics for four to six weeks is required. Surgery may be needed in patients with symptoms of heart failure due to valve dysfunction. Besides, early surgery is required for patients with complications such as large vegetations, paravalvular abscesses, or valve perforation, persistent bacteremia, or fever lasting longer than five to seven days after initiating appropriate antimicrobial therapy, or in patients with prosthetic valve endocarditis and relapsing infection [7]. Our patient was recommended to undergo surgery due to valve perforation, but she refused and was managed with antibiotics alone.

## Conclusions

This case highlights the importance of performing echocardiography for the early detection and appropriate management of IE in patients with streptococcal pneumoniae bacteremia. A high index of suspicion for bacterial endocarditis should be applied in all patients especially those with alcohol abuse, immunocompromised, previous valve disease, or cardiac anomalies. TTE has diagnostic limitations for the diagnosis of IE. TEE favors early detection of the disease and its complications and guides the appropriate therapeutic action throughout the clinical course. Treatment include prolonged course of intravenous antibiotics, and surgery may be needed in selected patients and was associated with better prognosis.
